# Do the data really support ordering fragile X testing as a first-tier test without clinical features?

**DOI:** 10.1038/gim.2017.64

**Published:** 2017-05-25

**Authors:** Veronique Weinstein, Pranoot Tanpaiboon, Kimberly A Chapman, Nicholas Ah Mew, Sean Hofherr

**Affiliations:** 1 Division of Genetics and Metabolism, Children’s National Health System, Washington, DC, USA; 2 Division of Laboratory Medicine, Children’s National Health System, Washington, DC, USA

**Keywords:** autism, chromosomal microarray analysis, fragile X syndrome, learning delay, intellectual impairment

## Abstract

**Purpose:**

Current guidelines recommend first-tier chromosome microarray analysis (CMA) and fragile X syndrome (FX) testing for males with isolated intellectual disabilities/learning delay (ID/LD) and autism spectrum disorders (ASDs).

**Methods:**

Males in our clinic with ID/LD or ASD (310) were analyzed for positive results from CMA and/or FX testing.

**Results:**

CMA detected abnormalities in 29% of males with ID/LD and only 9% of males with ASD (including variants of uncertain significance and absence of heterozygosity). When males with ID/LD were tested for FX, the detection rate was 2.5% (2 of 80). Both patients had dysmorphic features and maternal family history. No males with ASD had positive FX test results.

**Conclusions:**

The detection rate of CMA in males with isolated ID/LD in this study was higher than in the literature (10–20%). CMA results for males with ASD (9%) and FX testing for males with ID/LD (2.5%) overlap with the literature (7–10% and 2%, respectively). The yield of FX testing for patients with ASD was zero, which is close to that of the literature (0.5–2%). These results suggest that FX testing as a first-tier test may not be necessary, unless other criteria suggest FX.

## Introduction

Intellectual disability (ID) and autism spectrum disorders (ASDs) are relatively common and have a significant impact on the patient and their family. For this reason, identifying their possible genetic and environmental causes is important. ID has a prevalence of 3%^1, 2^ in the United States, with etiologies ranging from trauma to genetic disorders. A genetic basis has been demonstrated in multiple studies, and has been shown to be due to chromosome abnormalities, single genes, copy-number variants (CNVs) and multifactorial inheritance. The prevalence of ASD is 1 in 68 children or approximately 1.47% in 2012 per the Centers for Disease Control and Prevention (CDC).^3^ The heterogeneous phenotype of ASD includes autistic disorder, Asperger syndrome, and pervasive developmental disorder not otherwise specified (PDD-NOS) and occurs in all racial, ethnic, and social groups.^4^ It is characterized by impairments in social interaction, communication and language development, and rigid and repetitive behaviors^4^ with an onset before 3 years of age.^5^ The etiology for ASD in most cases is unknown, but in approximately 20–25% of patients it is found to be related to genetic changes or risks.^6^ The reported incidence of ID has increased significantly, while that of ASD has roughly tripled over the past 12 years,^3^ leading to an increased number of referrals to genetic clinics to attempt to find an etiology.^5^

Fragile X syndrome (FX) is considered the most common inherited cause of ID worldwide.^7^ In addition, FX is identified in 0.5–2% of ASD cases^4, 8, 9^ and 20% of boys with FX have a diagnosis of ASD.^10, 11^ FX is an X-linked disorder that in 99% of cases is due to a CGG-repeat expansion (>200 repeats) in the *FMR1* gene resulting in a penetrance of 100% in males. The main and most consistent feature of FX is ID, while physical and behavioral features vary with age and gender.^12^ The prevalence of males with a full mutation is ~1/4,000 to 1/7,000 in the total population,^13, 14^ and all ethnic groups and races appear to be susceptible to the expansion of the *FMR1* CGG region. FX testing has a diagnostic yield of ~2% in males and females with ID^15^ and ~0.46–5% for ASD. The FX test is unlikely to miscategorize an individual with a sensitivity of >99% and 100% specificity.

Chromosomal abnormalities are another common cause of ID/developmental delay (DD). Chromosomal microarray (CMA) provides a higher diagnostic yield^4, 15^ for genetic testing of patients with unexplained nondysmorphic ID/DD (10–20%) and ASD (~7–10%) than a G-banded karyotype (3%).^5, 16^

Therefore, CMA and FX testing are recommended by the American College of Medical Genetics and Genomics (ACMG) and the American Academy of Pediatrics as first-tier tests for individuals with ID or learning delay (LD) and ASD.^4, 5, 17, 18^

In our experience, FX is not as common in either ID/LD or ASD male populations as reported in the literature. Positive results were noted only in male patients with FX facial features and/or a family history of FX in siblings or a premutation mother (CGG repeats of 55–200). We hypothesized that the yield of FX testing is low and that CMA is a more sensitive test to provide a molecular diagnosis for such male patients, unless other criteria, including physical and psychological features and/or family history, are strongly suggestive of FX.

This study compares the molecular diagnostic yields of CMA and FX testing in two pediatric populations: males with ID/LD without ASD and males with ASD.

## Materials and methods

Data pertaining to all patient visits to the Children’s National Health System Genetics Department from 1 July 2013 to 19 February 2015 were collected, based on data from the billing department. This amounted to 10,757 charges. Of these, visits for patients with identifiable genetic syndromes, gross chromosomal differences (trisomies), and metabolic disorders were eliminated from the analysis, as were all charges for female patients.

These studies focused on two populations of male pediatric patients: (i) patients with isolated ID/LD (without ASD) and (ii) patients with ASD. Ages ranged from 14 months to 19 years. Males were chosen to increase the yield of possible positive testing results due to the X-linked nature of FX. All patients provided consent for their genetic testing, per institutional protocol.

### Patients with isolated ID/LD

**Figure 1:**
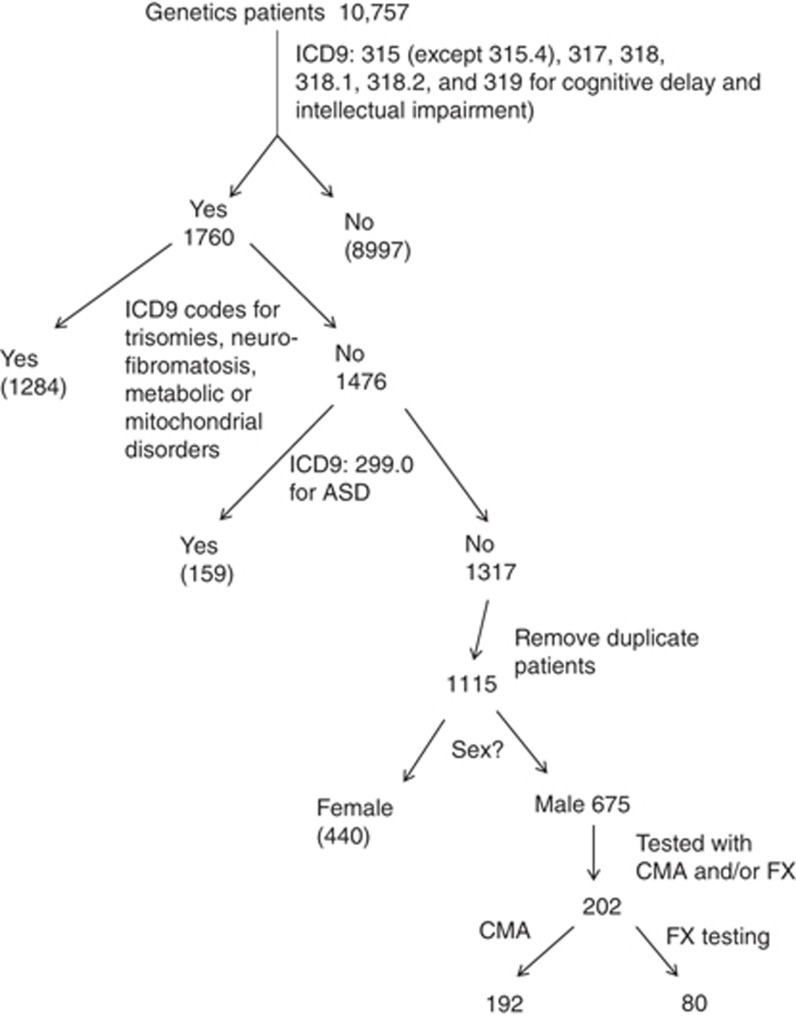
**Selection of pediatric male patients with ID/LD (and no diagnosis of ASD) who were tested by CMA and/or FX analysis.** ASD, autism spectrum disorder; CMA, chromosome microarray analysis; FX, fragile X; ICD, International Classification of Diseases, 9th revision; ID/LD, intellectual disabilities/learning delay; NF, no finding.

First, we identified 10,757 male patients with isolated ID who had undergone CMA and/or FX testing. Only those billed with at least one of the following International Classification of Diseases, 9th revision (ICD-9), codes were retained: under 315 (except 315.4 (coordination disorder)) for DD and 317 (mild ID), 318 (moderate ID), 318.1 (severe ID), 318.2 (profound ID) or 319 (mental retardation)). This left 1,760 charges in the pool. Among these, visits that were also charged with ICD-9 codes corresponding to metabolic disorders, trisomies, and neurofibromatosis were removed, leaving 1,476 charges of interest. Of these, those billed with the ICD-9 code 299.0 (autism disorder and PDD-NOS) were removed, leaving 1,317 charges of interest. Some of these charges corresponded to several visits of the same patient and duplicate charges were removed, leaving 1,115 patients of interest. Female patients were removed from that group, leaving 675 male patients. Of these, 202 were tested with CMA and/or FX testing. Figure 1 illustrates this selection process.

### Patients with ASD

**Figure 2:**
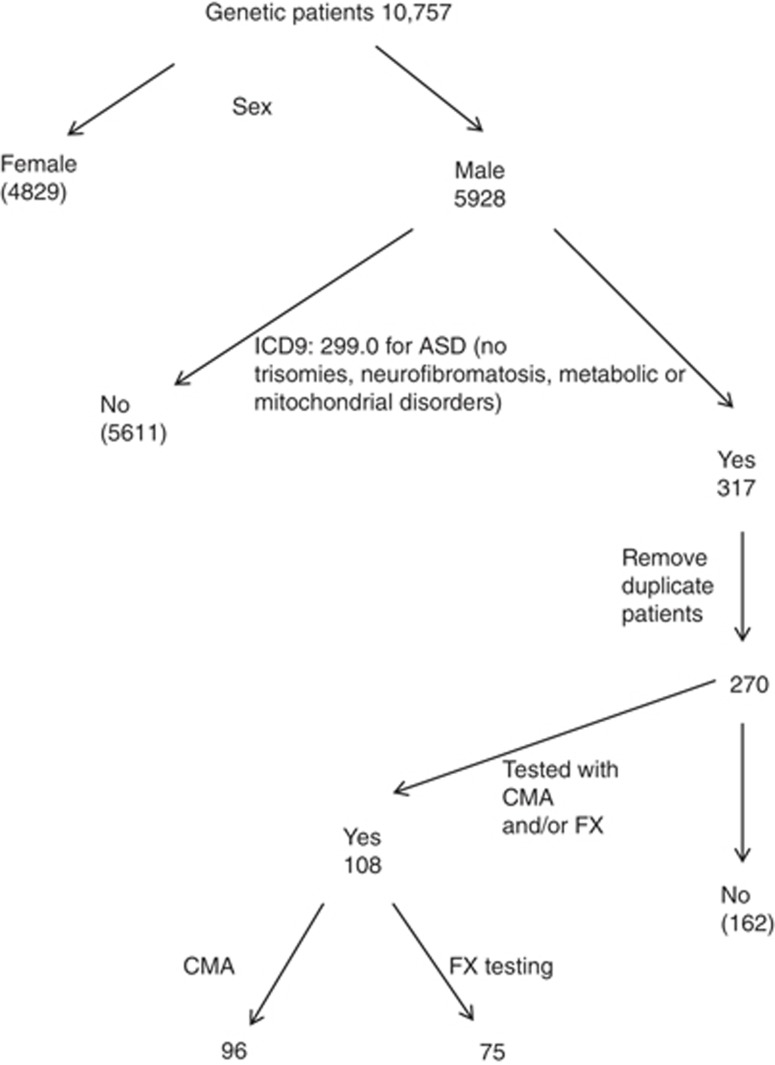
**Selection of pediatric male patients with ASD who were tested by CMA and/or FX analysis.** ASD, autism spectrum disorder; CMA, chromosome microarray analysis; FX, fragile X; NF, no finding.

To identify male patients with ASD who had undergone CMA and/or FX testing, the following approach was taken (Figure 2). From the 10,757 charges obtained from the billing department, only those billed for males were retained, leaving 5,928 charges. Among these 5,928 charges, those with the ICD-9 code 299.00 (autism disorder and PDD-NOS) were retained. Visits that had also been charged with ICD-9 codes corresponding to metabolic disorders, trisomies, and neurofibromatosis were removed, leaving 317 charges. Some of the remaining charges corresponded to several visits of the same patient. Duplicate charges were removed, leaving 270 patients of interest. Of these, 108 were tested with CMA and/or FX testing. Figure 2 illustrates this selection process.

### Clinical testing (CMA and FX testing)

The test results were collected and analyzed. The clinical notes from the geneticists who had evaluated the patients and ordered testing were reviewed for all patients whose CMA results were of uncertain significance or included areas of homozygosity. The photographs of the two patients diagnosed with FX were observed for clinical features.

CMA and FX testing were performed at the Children’s National Molecular Diagnostics Laboratory. For CMA, total genomic DNA isolated from peripheral leukocytes was analyzed for CNV and areas of homozygosity (AOH) by Affymetrix CytoScan Dx single-nucleotide polymorphism–based chromosomal microarray using the GeneChip System 3000Dx and by Chromosome Analysis Suite Dx Software (ChAS DX Software), approved by the US Food and Drug Administration.

For FX, a three-primer PCR amplification strategy (triplet-primed PCR) was performed to amplify the CGG repeat region in the 5′untranslated region of *FMR1*. Fragment analysis was then performed on an ABI 3130xl to determine the size of each allele’s repeat region. The fragment size was converted to number of repeats, which were reported using the ACMG Standards and Guidelines for Clinical Genetics Laboratories.

## Results

### CMA results of male patients with isolated ID/LD

Of the 202 male patients with ID/LD considered, 192 were tested by CMA; 56 had an abnormal CMA result with the potential to have caused the patients’ findings (28 of these had yet to undergo confirmatory testing). Twenty-eight (15%) of the 192 patients tested harbored pathogenic or likely pathogenic CNVs associated with their phenotype.

Of the 28 cases with abnormalities that were not pathogenic and required further evaluation, two patients harbored a likely pathogenic CNV (but it was unknown whether the CNV correlated with these patients’ phenotypes), and eight other patients harbored AOH (the autosomal recessive genes located in these areas had not yet been sequenced). Sixteen patients harbored CNVs categorized as variants of unknown clinical significance (VUS) and neither parent has been tested for their child’s VUS, limiting our ability to further interpret these variants. Two patients harbored CNVs categorized as VUS and had AOH with yet to be completed parental testing, making the interpretation of these VUS not possible. Sequencing performed on the autosomal recessive genes of the AOH was uninformative. Data are summarized in Table 1, which shows that the CMA sensitivity for 192 males diagnosed with ID/LD ranged from 15% (28/192) to 29% (56/192).

Of note, there were two patients who harbored AOH but were eventually diagnosed by another genetic test unrelated to CMA or FX testing. Two other patients, who are not listed in the table, harbored AOH, but sequencing of the autosomal recessive genes in these areas was negative.

### CMA results of patients with ASD

Of the 108 male patients with ASD considered, 96 were tested by CMA. Five of these (5%) harbored pathogenic or likely pathogenic CNVs associated with their phenotype. Two harbored AOH. The autosomal recessive genes located in these areas were not sequenced. Two further patients harbored CNVs categorized as VUS. Since no parental testing was completed, further interpretation of these VUS was not possible. It is possible that further investigation of these four cases would lead to a molecular diagnosis. The results are summarized in Table 2.

Thus, the CMA yield for 96 males diagnosed with autism ranged from 5 to 9%.

Of note, an additional patient harbored a pathogenic CNV; however, this was not known to be associated with autism or autistic features. Two other patients harbored AOHs and sequencing of the autosomal recessive genes located in these regions did not provide a diagnosis. Finally, two other patients harbored CNVs categorized as VUS but inherited from unaffected parents (one maternally, the other paternally), thereby decreasing the chance that these CNVs were causative of the patients’ autism.

### Fragile X results of males with isolated ID/LD

Of the 202 male patients with ID/LD considered, 80 were tested for FX. Two (2.5%) were positive, identified with fully expanded alleles. Both patients had physical findings and family histories concerning for FX.

The clinical characteristics of these two patients as indicated in their genetic clinic notes are reviewed below.

Patient 1 was first evaluated at 40 months when FX testing was recommended. At that time, he had frontal bossing, up-slanting palpebral fissures, long eyelashes of the upper and lower eyelids, bilateral epicanthal folds, large ears, DD, and autistic features (including repetitive behaviors, delayed nonverbal/problem-solving skills, and a history of social impairment, such as a lack of interest in playing with other children (although during the developmental assessment he showed moderate social interest)). He had not received a formal diagnosis of ASD. His maternal 7-year-old half-sister and a maternal uncle had been diagnosed with autism. His testing identified one fully expanded allele with more than 200 CGG repeats.

Patient 2 was first evaluated at 14 months of age when FX testing was recommended. He had slight frontal bossing with a recessed hairline, very large and fleshy ears with slight overfolded and squared-off helices, axial and peripheral hypotonia, and global DD. He was nonverbal and he had a tendency to propel himself out of caregivers’ arms and throw his body around the room, including onto the floor when unhappy. His family history showed mild learning differences in the males on the maternal side of his family. He was found to have size mosaicism for FX with more than 200 CGG repeats and a premutation size of 146 CGG repeats.

### Fragile X test results of patients with ASD

Of the 108 male patients with ASD considered, 75 were tested for FX. All had a negative result.

## Discussion

In summary, the yield of CMA measured by this study in pediatric males with ID/LD of 15–29% is slightly higher than that reported in the literature of 10–20% (Table 3). The yield of CMA measured by this study in pediatric males with ASD of 5–9% overlaps that reported in the literature of 7–10%. The yield of FX testing measured by this study in pediatric males with LD/ID of 2.5% is close to that in the literature of 2%. The yield of FX testing measured by this study in pediatric males with ASD of 0% is lower than that reported in the literature of 0.5–2% (Table 3). Here, we demonstrate that CMA provides more diagnoses than FX testing in males with ID/DD or ASD. Moreover, in the two patients with positive FX testing, there was a strong suspicion of FX before molecular testing, based on their facial features and family history of DD/LD/autism in several individuals on the maternal side of the family. This brings to question whether FX testing should be done as a first-tier test for DD/ID and in the absence of physical findings or a family history, or rather if it should be a second-tier test.

The increased number of ID/LD and ASD diagnoses has led to higher demand for CMA and FX testing, both of which are recommended by the CDC, American Academy of Pediatrics and ACMG as first-tier evaluations.^18^ There is no recent publication regarding the diagnostic yield of FX testing for patients with ID and/or ASD. Since the yield of FX testing is low, we suggest screening potential candidates for FX before ordering the test, as opposed to systematically ordering FX testing as a first-tier test. Future research could include establishing a pre-test checklist to predict the possibility of a positive FX result. As previously reported in the literature, the use of physical and psychological checklists by a geneticist or dysmorphologist to select patients with a high probability of FX may reduce the number of individuals who are submitted for molecular evaluation by 60–80%.^19^ Age-appropriate (pre- versus postpubertal) checklists could include a family history of ID or ASD, an elongated face, large ears, hyperextensible finger joints, large testes, a plantar crease, hand biting, hand flapping, tactile defensiveness, poor eye contact, brain anomalies, and others.^15^ Since this study was done in our genetics clinic, clinicians are comfortable with identifying physical findings consistent with FX, which may have influenced the testing selection. Checklists may assist the nongeneticist in determining whether this testing is indicated.

Since genetic testing is expensive and, from our experience, insurance may cover only one test, CMA should have precedence over FX testing, unless further clues point to FX. As always, good history taking, including family history and physical exam, are the cornerstone of making a diagnosis in these patients.

In our study, the diagnostic yield of CMA for male patients with isolated ID/LD was higher than previously reported. This may be related to the fact that our population was all male and there is a higher prevalence of ID/LD in males. According to the CDC, males have twice the prevalence of any DD than females and, more specifically, have a higher prevalence of attention deficit hyperactivity disorder, autism, learning disabilities, stuttering/stammering, and other DDs. This discrepancy is probably also related to the nature of our practice (nonreference testing with a large cadre of geneticists) and to the fact that VUS were not excluded.

FX testing is relatively inexpensive and there are currently no clinically available therapies, in contrast to some disorders diagnosed by chromosomal microarray and whole-exome sequencing. Whole-exome sequencing has a growing role in genetic evaluation, but CMA is also important.^20, 21^ Regardless of the ultimate diagnosis and testing regimen, inappropriate selection of testing can lead to a diagnostic odyssey, which can be expensive and frustrating. Although there are no clinically available interventions for individuals with FX, there are several ongoing trials for therapies; therefore, since FX testing is relatively inexpensive, even with a lower yield compared with CMA and whole-exome sequencing, there continues to be a role for it in appropriately selected patients, especially because diagnosis is also important for reproductive counseling, neurological and endocrine follow-up, and treatment for female carriers due to the increased risk of primary ovarian insufficiency.

We grant that there are several limitations of this study, For example, we relied on ICD-9 codes for the selection of our patient population. The ICD-9 codes were assigned by a number of geneticists at the Children’s National Health System. The consistency of selection of the ICD-9 codes between providers was not checked. At least one patient who did not have ASD but did have delay was assigned the 299.0 ICD-9 code for autism. This error was noticed while exploring the results. That patient was removed from the autism group and counted as a patient with cognitive impairment. It is possible that other errors passed unnoticed. We aimed to limit our studies to males with isolated ID/LD or ASD by using selected ICD-9 codes. In addition, we could not guarantee that those within the isolated ID/LD groups did not have syndromic features due to our approach. Therefore, it is possible that males with additional phenotype findings were included in the study.

In conclusion, early genetic diagnosis in males with ID/LD and ASD is important as it may give them access to earlier interventions (e.g., special educational services) and consequently improves outcome. Additionally, it enables providers to explain recurrence risks to the family beyond those of multifactorial inheritance, while sparing the family the burden of a costly diagnostic odyssey. As genetic test coverage by insurance is limited, it is important to select first-tier tests with the highest diagnostic yield. The results of this study align with those reported in the literature regarding the considerably higher yield of CMA compared with FX testing for males with ID/LD or ASD. Further studies need to be done to verify these results; however, we suggest that unless other criteria are considered in addition to ID/LD or ASD, performing FX testing as a first-tier test in males may not be necessary; rather, it is better suited in this situation as a second-tier test. We also suggest the use of pretest, age-dependent algorithms to help screen patients who should undergo FX testing, to avoid missing patients.

## Figures and Tables

**Table 1:** Summary of chromosome microarray analysis results in males with intellectual disability (no autism)

**Description**	***n*****(%)**
Total male patients with ID/LD	192
Pathologic or likely pathologic CNV	28 (15)
Likely pathologic CNV but unknown in this phenotype	2 (1)
AOH, no sequencing completed on the autosomal recessive genes in this region	8 (4)
VUS; no parental testing	16 (8)
VUS and AOH; no parental testing, and sequencing not informative	2 (1)

AOH, absence of heterozygosity; CNV, copy-number variant; ID/LD, intellectual disabilities/learning delay; VUS, variant of uncertain significance.

**Table 2:** Summary of chromosome microarray analysis results in males with ASD

**Description**	***n*** **(%)**
Total male ASD patients	96
Pathogenic or likely pathogenic CNVs	5 (5)
AOH, but autosomal recessive genes in this region not sequenced	2 (2)
CNVs that were VUS; no parental testing done	2 (2)

ASD, autism spectrum disorder, AOH, absence of heterozygosity; CNV, copy-number variant; VUS, variant of uncertain significance.

**Table 3:** Comparison of CMA and Fragile X test results between patients with isolated ID/LD and patients with ASD

**Population**	**CMA sensitivity**	**Fragile X test sensitivity**
ID/LD (no ASD)	14.5–29% (28/192–56/192)	2.5% (2/80)
ASD	5–9.5% (5/96–9/96)	0% (0/75)
Total	11.5–22.5% (33/288–65/288)	1.5% (2/155)

ASD, autism spectrum disorder; CMA, chromosome microarray analysis; ID/LD, intellectual disabilities/learning delay.

## References

[bib1] Petersen MC, Kube DA, Palmer FB. Classification of developmental delays. Semin Pediatr Neurol 1998;5:2–14. 9548635 10.1016/s1071-9091(98)80012-0

[bib2] Michelson DJ, Shevell MI, Sherr EH, Moeschler JB, Gropman AL, Ashwal S. Evidence report: genetic and metabolic testing on children with global developmental delay: report of the quality standards subcommittee of the American academy of neurology and the practice committee of the child neurology society. Neurology 2011;77:1629–1635. 21956720 10.1212/WNL.0b013e3182345896

[bib3] Boyle CA, Boulet S, Schieve LA et al, Trends in the prevalence of developmental disabilities in US children, 1997-2008. Pediatrics 2011;127:1034–1042. 21606152 10.1542/peds.2010-2989

[bib4] Shen Y, Dies KA, Holm IA et al, Clinical genetic testing for patients with autism spectrum disorders. Pediatrics 2010;125:e727–e735. 20231187 10.1542/peds.2009-1684 PMC4247857

[bib5] Schaefer GB, Mendelsohn NJ. Clinical genetics evaluation in identifying the etiology of autism spectrum disorders: 2013 guideline revisions. Genet Med 2013;15:399–407. 23519317 10.1038/gim.2013.32

[bib6] Miles JH. Autism spectrum disorders—a genetics review. Genet Med 2011;13:278–294. 21358411 10.1097/GIM.0b013e3181ff67ba

[bib7] Gallagher A, Hallahan B. Fragile X-associated disorders: a clinical overview. J Neurol 2012;259:401–413. 21748281 10.1007/s00415-011-6161-3

[bib8] Roesser J. Diagnostic yield of genetic testing in children diagnosed with autism spectrum disorders at a regional referral center. Clin Pediatr (Phila) 2011;50:834–843. 21525079 10.1177/0009922811406261

[bib9] McGrew SG, Peters BR, Crittendon JA, Veenstra-Vanderweele J. Diagnostic yield of chromosomal microarray analysis in an autism primary care practice: which guidelines to implement? J Autism Dev Disord 2012;42:1582–1591. 22089167 10.1007/s10803-011-1398-3

[bib10] Clifford S, Dissanayake C, Bui QM, Huggins R, Taylor AK, Loesch DZ. Autism spectrum phenotype in males and females with fragile X full mutation and premutation. J Autism Dev Disord 2007;37:738–747. 17031449 10.1007/s10803-006-0205-z

[bib11] Hatton DD, Sideris J, Skinner M et al, Autistic behavior in children with fragile X syndrome: prevalence, stability, and the impact of FMRP. Am J Med Genet A 2006;140A:1804–1813. 16700053 10.1002/ajmg.a.31286

[bib12] Hagerman RJ, Amiri K, Cronister A. Fragile X checklist. Am J Med Genet 1991;38:283–287. 2018072 10.1002/ajmg.1320380223

[bib13] Hill MK, Archibald AD, Cohen J, Metcalfe SA. A systematic review of population screening for fragile X syndrome. Genet Med 2010;12:396–410. 20548240 10.1097/GIM.0b013e3181e38fb6

[bib14] Coffee B, Keith K, Albizua I et al, Incidence of fragile X syndrome by newborn screening for methylated FMR1 DNA. Am J Hum Genet 2009;85:503–514. 19804849 10.1016/j.ajhg.2009.09.007 PMC2756550

[bib15] Christofolini DM, Abbud EM, Lipay MV et al, Evaluation of clinical checklists for fragile X syndrome screening in Brazilian intellectually disabled males: proposal for a new screening tool. J Intellect Disabil 2009;13:239–248. 19786505 10.1177/1744629509348429

[bib16] Miller DT, Adam MP, Aradhya S et al, Consensus statement: chromosomal microarray is a first-tier clinical diagnostic test for individuals with developmental disabilities or congenital anomalies. Am J Hum Genet 2010;86:749–764. 20466091 10.1016/j.ajhg.2010.04.006 PMC2869000

[bib17] Sherman S, Pletcher BA, Driscoll DA. Fragile X syndrome: diagnostic and carrier testing. Genet Med 2005;7:584–587. 16247297 10.1097/01.GIM.0000182468.22666.dd PMC3110946

[bib18] Moeschler JB, Shevell M. Comprehensive evaluation of the child with intellectual disability or global developmental delays. Pediatrics 2014;134:e903–e918. 25157020 10.1542/peds.2014-1839 PMC9923626

[bib19] Mandel JL, Biancalana V. Fragile X mental retardation syndrome: from pathogenesis to diagnostic issues. Growth Horm IGF Res 2004;14(suppl A):S158–S165. 15135801 10.1016/j.ghir.2004.03.034

[bib20] Yang Y, Muzny DM, Xia F et al, Molecular findings among patients referred for clinical whole-exome sequencing. JAMA 2014;312:1870–1879. 25326635 10.1001/jama.2014.14601 PMC4326249

[bib21] Posey JE, Harel T, Liu P et al, Resolution of disease phenotypes resulting from multilocus genomic variation. N Engl J Med 2017;376:21–31. 27959697 10.1056/NEJMoa1516767 PMC5335876

